# Microplastics profile along the Rhine River

**DOI:** 10.1038/srep17988

**Published:** 2015-12-08

**Authors:** Thomas Mani, Armin Hauk, Ulrich Walter, Patricia Burkhardt-Holm

**Affiliations:** 1 Man-Society-Environment (Programme MGU), Department of Environmental Sciences, University of Basel, Vesalgasse 1, 4051 Basel, Switzerland; 2 Intertek, Switzerland, 4002 Basel; 3 Department of Biological Sciences, University of Alberta, Edmonton, Canada

## Abstract

Microplastics result from fragmentation of plastic debris or are released to the environment as pre-production pellets or components of consumer and industrial products. In the oceans, they contribute to the ‘great garbage patches’. They are ingested by many organisms, from protozoa to baleen whales, and pose a threat to the aquatic fauna. Although as much as 80% of marine debris originates from land, little attention was given to the role of rivers as debris pathways to the sea. Worldwide, not a single great river has yet been studied for the surface microplastics load over its length. We report the abundance and composition of microplastics at the surface of the Rhine, one of the largest European rivers. Measurements were made at 11 locations over a stretch of 820 km. Microplastics were found in all samples, with 892,777 particles km ^−2^ on average. In the Rhine-Ruhr metropolitan area, a peak concentration of 3.9 million particles km ^−2^ was measured. Microplastics concentrations were diverse along and across the river, reflecting various sources and sinks such as waste water treatment plants, tributaries and weirs. Measures should be implemented to avoid and reduce the pollution with anthropogenic litter in aquatic ecosystems.

Our environment is increasingly polluted by plastic debris, which is found on beaches, surface waters, and the marine benthos worldwide^1^. Microplastics fragments (<5 mm) are ingested by aquatic organisms, with negative consequences for survival, fitness, reproductive output and health^1^,^2^,^3^. Microplastics are apparently transferred from one biological level to the next^4^. They also contain a multitude of chemical additives such as antioxidants, processing chemicals, colorants and pigments and adsorb hydrophobic contaminants from the surroundings^5^.

Microplastics pollution was first recognized in marine ecosystems^6^. An estimated 80% of plastic in the sea originates from inland sources and is emitted by rivers to the oceans^7^. The quantitative contribution of rivers as sources of microplastics has yet to be established^8^. So far, few freshwater systems have been investigated in this respect and little quantitative data are available on the amounts and distribution, categories and polymer types of microplastics in rivers^9^,^10^,^11^.

The Rhine is one of Europe’s major rivers and enters the North Sea through the Rhine-Meuse Delta near Rotterdam. The North Sea is highly polluted with microplastics^6^, and large rivers such as Thames and Rhine contribute to this pollution^12^,^13^. The Rhine has been rectified and regulated since the mid-19^th^ century, and in the upstream stretch (between Basel and Mainz) it is backed-up. The banks are often fortified and protected by groins and rip-raps. Six countries border this river and its catchment is characterised by high population densities. Fifty million people inhabit the catchment and 10% of the world’s chemical industry is situated here. In addition, very many textile, metal and plastics plants are located along the river. North-Rhine Westphalia alone is home to around 1,000 companies in the plastic industry with approximately 118,600 employees (about 22% of German companies in this sector)^14^. The pollution with many anthropogenic chemicals has been intensely investigated for many years in the Rhine River, but the plastic load has not yet been studied^15^.

We provide a first investigation of floating microplastics particles along the Rhine with respect to the quantity along the 820 km stretch between Basel and Rotterdam and their distribution along and across the river. The geographical framework is defined by Basel and Rotterdam being the first/last sampling sites and the first/last major industrial cities along the Rhine, respectively. The commercial shipping route starts and ends in Basel, and Rotterdam is the largest port of Europe before this river enters the North Sea. We hypothesized higher concentrations downstream of cities with high population density and industrial sites (Strasbourg, Mainz, Rhine-Ruhr area) as well as at the entry of highly polluted tributaries (at Mainz: Main River), and a lower concentration at sites with lower population density (e.g. Seltz/Rastatt). Sampling across the river is expected to provide insight into the effect of possible microplastics sources at either side of the respective sampling location (e.g. inflows of WWTP and tributaries; Supplementary Table 1).

We further assumed that the composition of microplastics in terms of particle morphology and polymer types would give indications on their origin and (former) use. We concentrated on the detection of light-weight microplastics of low specific density (<1 g cm^−3^) for the following reasons: They comprise common varieties of polyethylene and polypropylene, and partly also polystyrene (≤1.05 g cm^−3^). These types of plastics account for 55.7% of the annual demand in the EU and are used for a wide range of applications such as packaging, office equipment, and vehicle construction^16^. Along the Rhine, the vehicle construction and supply industry is concentrated, as are many large packaging companies and office furniture firms. Light-weight plastics tend to remain buoyant, floating on the surface and travelling long distances^1^,^5^. Finally, they can be sampled with a standardized surface sampling method. We therefore expect to find the bulk of microplastics on the water surface originating from light-weight materials.

## Results and Discussion

**Figure 1:**
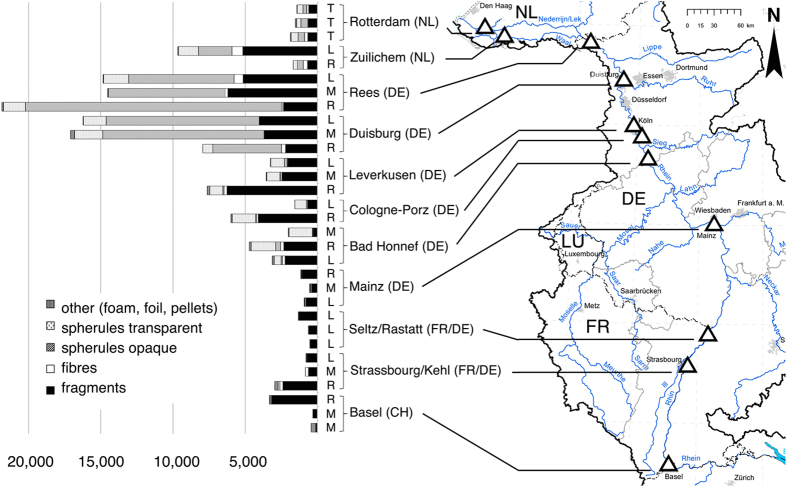
Number of microplastic particles (300 μm−5 mm) 1000 m^−3^ in categories at all sampling sites (Δ). The horizontal columns present microplastic abundance 1000 m^−3^ and the respective fraction of categories. *L*: *left bank*, M: *mid-river*, R: *right bank*, T: *transect* (position in the river cross section). The figure was created using Adobe Photoshop CS4, Version 11.0.2 to assemble the columns (Microsoft Excel for Mac 2011, Version 14.4.8) and the map (intern map by the ICPR Secretariat, 2011^44^; modified).

Microplastics particles were found in all samples (Fig. 1, Supplementary Tables 1–3). The 31 samples at 11 locations yielded 25,956 microplastics particles for a water surface of 25,745 m^2^. The total volume of water filtered was approximately 4,634 m^3^. This resulted in a weighted average of 892,777 particles km^−2^.

Our results reflect an accumulation of the discharges, number of inhabitants, urban centres and industrial plants over the river stretch, increasing the microplastics load downriver^5^. There is an ascending trajectory of concentrations from the minimum averages (Basel - Mainz) via medium averages (Bad Honnef, Cologne-Porz and Leverkusen) to the highest averages in the Rhine-Ruhr metropolitan area. At Rees, we documented the peak of 3.9 million particles km^−2^ in a single sample (Fig. 1 and Supplementary Table 2). The six samples at Rees and Duisburg accounted for more than 66% of all microplastics recovered during the whole sampling campaign. This supports findings of higher microplastics concentrations near densely populated areas^17^,^18^.

The heterogeneity of the microplastics concentration at the sampling sites along and across the river is caused by several factors such as proximity of waste water treatment plants (WWTPs), mixing of outlet plumes, hydraulic peculiarities (e.g. ship traffic), turbulences, geomorphological characteristics and, last but not least, non-continuous releases, seasonal and weather events. Clearcut correlations, however, are rarely obvious because the interaction between microplastics particles and the likely responsible factors are driven by hydrological dynamics and are thus highly complex^13^,^19^.

WWTP outlets are important point sources of microplastics^5^,^18^,^20^,^21^. Similarly, some of the considerable differences in densities at our sampling sites could be explained by the impact of WWTP outlets (Supplementary Tables 1–3). Seven out of eleven of our sampling sites exhibited concentrations in the range between the upstream and downstream values of the highly urbanized North Shore Channel in Chicago, Illinois, USA. This channel contained 17,930 microplastics particles 1,000 m^−3^ (or 6,698,264 km^−2^) downstream and 1,940 items 1,000 m^−3^ (730,341 km^−2^) upstream of such an outlet (333 μm mesh)^9^. Five of our sampling sites with such high concentrations are in the Rhine stretch in North-Rhine Westphalia, the most densely populated federal state in Germany (except city states), inhabited by more than 10 million people (>1,000 km^−2^) in the Rhine-Ruhr area alone^22^. Here, 406 WWTPs line the Rhine and its tributaries, treating the sewage of an equivalent of 20.7 million inhabitants^23^.

Exceptions to the general ascending trajectory along the Rhine must be acknowledged as well. These can be due to sinks and retention of particles, to turbulences, stillwaters and drift to the river banks where particles are washed ashore^13^. Along the sampled river stretch in the Upper Rhine valley (Basel-Mainz), 8 weirs back-up the river, acting as sinks for light-weight particles (24, personal communication A. Schmidt).

Most prominent and unexpected is the drop in concentrations at Zuilichem (right bank) and in the Rhine-Meuse Delta towards Rotterdam. Here, a major microplastics retention process may be taking place. This is likely because the Rhine has its lowest slope here, slowing flow velocity, whereby sedimentation rates increase for particles with a specific density similar to water. This could be enhanced when microplastics are settled by fouling organisms, increasing the overall density^25^,^26^. Experiments have shown that this effect first becomes relevant after one week or more^27^. The only sampling location at we expect this effect is in the lower Part of the Rhine-Meuse Delta near Rotterdam where, due to tidal dynamics and low discharge, the exposition duration of microplastics could be sufficient for biofouling and subsequent sedimentation. Sedimentation could additionally be enhanced by the tidal exchange with heavier, brackish water.

Across the river, concentrations differ as well. This is partly due to turbulences and partly due to entry of effluents of industries, treated wastewater or polluted tributaries. Thus, the sampling site at Basel 3, right bank, is probably influenced by the outlet of the WWTP of Basel, releasing its effluents at this area and this river side. At Mainz, the Main River enters the Rhine at the right river bank, leading to a higher concentration than in the middle and on the left bank. This is in line with data on the microplastics concentration at the right shores of the Rhine after the confluence with the Main^13^. Even higher concentrations might be expected after the confluence with the polluted Main, but a sluice shortly before the confluence act as sedimentation trap for the particles (A. Schmidt, personal communication). The higher concentrations at the right river bank versus the middle and left bank at Cologne-Porz and Leverkusen are possibly due to WWTP effluents entering the Rhine from the orographically right side, just upstream of the sampling site.

The concentrations of microplastics particles in the Rhine are much higher than in most of the studies on river surfaces and lakes^28^. For the most polluted Swiss lakes, Lake Geneva and Lake Maggiore, 220,000 items km ^−2^ were reported^17^. Values for other lakes were 105,503 km ^−2^ (Lake Erie), 5,390 km ^−2^ (Lake Superior), 2,779 km ^−2^ (Lake Huron) and 20,000 km ^−2^ (Lake Hovsgol)^18^,^29^. In this study as well as in that on Swiss water bodies^17^, the mesh size of the Manta net was 300 μm, while in the studies of the Great Lakes and the Mongolian Lake it was 333 μm^18^,^29^, potentially yielding somewhat higher numbers in our study.

We found different categories of microplastics (see Supplementary Fig. 1), including opaque spherules (45.2%), fragments (37.5%), transparent spherules (13.2%), fibres (2.5%) and others (1.1%). Spherules are manufactured plastic products used in feedstock for the plastic industry, or as scrubbing granules and pellets in cosmetic products, in air-blasting agents or in industrial cleaner and other products^30^. Microplastics fragments and fibres result from fragmentation or abrasion and weathering of larger plastic items^8^. The FT-IR spectroscopy of 118 samples revealed polystyrene (29.7%) as the dominant polymer, followed by polypropylene (16.9%), other types (13.6%), acrylate (9.3%), polyester (5.1%) and polyvinyl chloride (1.7%). Thus, 86.4% of all particles analysed were identified as being among the worldwide most produced polymers. The remaining 13.6% particles could not be assigned to a specific type of polymer, though the spectra indicated the presence of typical plastic additives associated with the particles.

**Figure 2:**
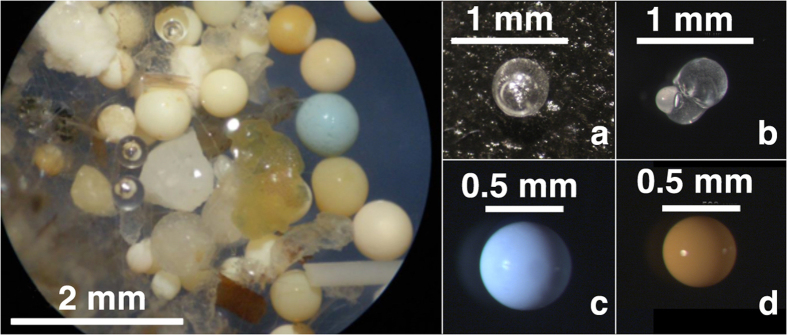
Typical microplastic categories in the Rhine. Left: Duisburg sample consisting of 65% opaque spherules, further fragments and fibres, bar: 2 mm. (**a/b**) transparent spherules with gas bubbles, polymethyl-methacrylate (Zuilichem), bars: 1 mm; (**c/d**) opaque spherules, polystyrene (Duisburg, Rees), bars: 500 μm.

In our study, spherules made up almost 60% of the total findings. The majority were opaque with a smooth surface, a spherical shape, and a size range of 300–1000 μm (Fig. 2 and Supplementary Fig. 2). Almost 70% consisted of cross-linked polystyrene, and 15% of the samples we measured were made of polyethylene (Supplementary Fig. 1). These spherules occurred in substantial numbers only downstream from Duisburg to Zuilichem. The polystyrene spherules possibly originated from industrial processes such as air-blasting^31^ or from the plastic waste that manufacturers washed into the water treatment systems^32^. Plastic manufacturers are located in high numbers along the Rhine and in close proximity to the named sampling sites in North Rhine Westphalia (Supplementary Fig. 1)^14^. These particles were reported to be especially common in the environment near plastic processing plants and the sewage plants treating their waste water ^32,33^. The high numbers (Duisburg: 8,848 particles 1,000 m^−3^; Rees: 11,050 particles 1,000 m^−3^) correspond to data from the Austrian Danube, where pellets were measured at a mean density of 693 items 1,000 m ^−3^ (maximum: 138,219 items 1,000 m^−3^)^10^. The latter values were due to losses at a plastic pellet production site during a heavy rainfall event^33^.

The opaque spherules composed of polyethylene may have originated from personal care products because this material is a common ingredient^34^.

Most of the transparent spherules measured 400–900 μm in diameter. These spherules often occurred in small aggregates of different sizes and the individual items occasionally contained air bubbles (Fig. 2a,b). They are composed of polymethyl methylacrylate (PMMA, 85%) or polystyrene (15%) and first appeared in samples at Bad Honnef and continued to be found all the way to The Netherlands. This type of spherules is used as raw material for construction, aviation, furniture, lighting and electronics^35^. With a specific density of 1.17 g/mL, pure forms of PMMA are expected to sediment in freshwater. The inclusion of gas bubbles within the PMMA spherules, consuming up to 30% of the total spherule volume in the Rhine samples, probably explain the buoyancy.

Microplastics from secondary sources tend to be associated with sites of higher population densities^5^. Fragments and fibres are distributed very heterogeneously across the river at each of our sites and between the sites along the river. This indicates that local sources and hydrological conditions are responsible for these variations. In contrast to earlier studies, it is unlikely that most fibres in our samples originated from clothes: in our samples 68.4% of this fraction was composed of polypropylene, whereas synthetic fibres from clothes consist mostly of polyester and acrylic fibres^36^.

Our findings demonstrate considerable pollution of the Rhine with microplastics. Estimating the load that the Rhine contributes to the North Sea is, of course, preliminary, given the snap-shot character of our study. Nevertheless, we extrapolated the mean microplastics concentration exported to the North Sea. Downstream of Rees, the river splits up into the Nederhijn and the Waal with the city Zuilichem. On average, 64–68% of the original Rhine discharge passes the Waal^37^. Because the Rhine splits into numerous arms in the Rhine-Meuse Delta, the values measured in Rees are a better indication for the export of microplastics by the river to the North Sea than Zuilichem, Rotterdam, or the sum of both.

Estimating the mean microplastics concentration on the day of sampling in Rees, a daily freight of over 191.6 million microplastics particles at and beneath the surface are discharged towards the North Sea within the reach of a Manta Trawl sampling (0.18 m depth; the figure is calculated by multiplying the length of water passing the sampling location in 24 hours (148,953.6 m) * the river width (419 m) * the Manta Trawl depth (0.18 m) * mean microplastics particle concentration (17.061 # m^−3^). This is a conservative approach since it neglects the suspended and bottom-near particles. However, a spot test with 50,000 litres of Rhine water at approximately 5 m depth in Basel (Rhine Survey Station) showed no measurable microplastic particles (Mani, unpublished data). This finding supports the focus towards the water surface when calculating daily freight discharges.

In conclusion, the high average concentration, the high contribution of over 191 million items of microplastics alone floating on the river surface, and the extremely high concentrations of spherules highlights the important contribution of this river to the microplastics mass in the North Sea. It has to be studied how the pollution with microplastics can effectively be reduced, under consideration of the whole ‘life cycle’ of anthropgenic litter, i.e. different sources and entry pathways for primary plastic particles (spherules, pellets: probably directly released from plants or indirectly via run-offs) and for secondary microplastics (e.g. fragments and fibres via sewage overflow and waste water effluents)^38^. In light of our results, we emphasize the importance and urgency of immediate measures in the management of plastic debris. So far, legal implementation in most European countries is largely missing or insufficient^27^,^33^. New frameworks might improve legislation: for example, the aim of the German marine policy is a comprehensive management of human activities to achieve a good status of marine waters by 2020^39^. Under these auspices, the focus is on reducing inputs of litter through a combination of measures relating to product design, waste management, aftercare and public awareness raising. More specifically, this ‘Programme of Measures’ propose the following: avoiding the use of primary microplastics; reducing inputs of plastic litter, e.g. plastic packaging, into the marine environment; reducing amounts of plastic litter through local regulatory provisions; reducing emissions and input of microplastic particles^39^. We suggest to implement the measures for freshwater environments in countries with microplastics pollution as well.

## Methods

### Sample collection, preparation and processing

Thirty-one samples were collected at 11 sites (Supplementary Table 1) from surface waters during June to July 2014 using a Manta net (rectangular opening of 60 cm x 18 cm, 300 μm mesh)^17^. Sampling sites were selected according to the following criteria: first sampling site Basel, the city with the most upstream commercial harbour, and last sampling site is Rotterdam, the city with most downstream harbour (to encompass shipping route), big cities with high number of inhabitants and industry (Strasbourg, Seltz/Rastatt, Mainz, etc.) and an even distribution between the first and last sampling site (Supplementary Table 1).

A flowmeter (HYDRO-BIOS, model 438 110) was employed at the center of the Manta opening to quantify the sampled surface area and volume. In contrast to marine surface samplings^1^, quantifying the volume was possible (volume Manta: 0.108 m^3^). Wave dynamics and trawling velocity variation were negligible in the Rhine and thus the whole Manta aperture faced inflowing water during each entire sampling process.

The Manta was deployed from vessels at 10 of the 11 sampling locations. In four locations the Manta was towed to the back of an upstream-facing vessel (Basel, Strassbourg/Kehl, Bad Honnef and Zuilichem). In Rotterdam, samples were taken by trawling the Manta through the water. At the remaining five locations the net was deployed at the side of the vessel with a gap of 2–4 m to avoid turbulence caused by the bow (Mainz, Cologne-Porz, Leverkusen, Duisburg and Rees). At Seltz/Rastatt, the device was attached to the pillar of a riverside ferry pier construction. It was oriented to sample in the middle, at the right and the left quartile of the river cross section (Supplementary Table 1). In Seltz, only the left quartile and in Basel only the middle quartile were sampled. Sampling time was 15 minutes each. With an average river flow velocity of 1.4 m/s over all sampling sites (exception Rotterdam) a 15-minute sample is equivalent to an 11-minute tow by boat in Rotterdam at a speed of 3.5 knots^29^. 15-minute samples yielded filtered water volumes of 60–250 m^3^ (mean 150 m^3^). The total number of particles was expressed as weighted average per km^−2^. The weighted average was calculated by taking the average concentration in every sampling location and weighing it down by multiplying it with the number of samples taken in that location. The final figure was then divided by the total number of samples (n = 31). This way the total number of samples did not get lost within the average calculation despite there not being an equal number of samples taken at each location along the river.

Samples were transferred into a glass beaker by rinsing with local tap water and subsequently stored in 10% NaCl in glass jars for conservation. To reduce the risk of sample contamination by artifacts such as clothing fibres during on-board recovery, samples were held against the wind to avoid any airborne contamination. In the laboratories, samples were first run through a stacked series of metal sieves (5 mm, 1 mm and 300 μm) with Milli-Q water. Pieces of biological organic material sized >5 mm were rinsed and discarded. All fractions were kept at 5 °C.

To remove material of biological organic origin such as leaves, grass, or shells, the >300 μm fractions were enzymatically treated (Löder, M.G.J., personal communication). For this, fractions were first treated in 0.25% sodium dodecyl sulfate (SDS) in Milli-Q water (pH 7.9–8.1), then stirred at 450 rotations min ^−1^ for 10 minutes and afterwards incubated at 70 °C overnight. After the samples cooled to 40 °C, they were incubated with 0.5% each of Biozym F (lipase) and SE (protease, amylase) for three days at 37 °C. Afterwards, the samples were sieved and rinsed with Milli-Q water on a 300 μm mesh. Material of biological origin was further degraded through a hydrogen peroxide oxidation (30% H_2_O_2_) at 37 °C for 24 h^40^,^40^. Rinsing with Milli-Q water was followed by treatment with 10% chitinase in phosphate buffer (pH 5.6) for 5 days at 37 °C and, after rinsing again, followed by incubation with 10% cellulase for 24 h at 50 °C. Samples went through a salinity-based density separation using sodium chloride (23% NaCl/water; density 1.16 g cm^−3^)^6^,^41^. Additionally, a drop of detergent was added to break the surface tension. After 4 h at room temperature, the heavier material was drained from the sample.

Samples were rinsed on a 300 μm mesh and transferred into a Bogorov chamber (HYDRO-BIOS, Altenholz, Germany, 140 × 80 mm, 70 ml) under a stereomicroscope with super- and sub lighting capacity. The sample was then carefully shifted along the meander of the Bogorov chamber and scanned for potential plastic components visually. Visible putative plastic particles were collected for further analysis, pre-distinguished by several criteria indicating a hydrocarbon polymer compound: colorful and homogeneous particles with no visible biological cellular structure; particles with a homogeneous sphere (spherule) or thickness (fibre). Further, plastic particles can usually be dented in with tweezers but not easily cut. Hard plastic would break under the same pressure. Detected microplastic particles were counted and sorted into categories: fibres, fragments, spherules transparent, spherules opaque, and foam. The selection of particles for spectroscopic analysis (n = 118) was designed to cover putative plastic particles from a) every sampling location as well as b) from every category found (fragments, fibres, spherules, etc.).

### Identification and quantification

We performed a Fourier transform infrared spectroscopy (FT-IR) on the most frequent particle categories to validate microplastics identification and facilitate comparison with other studies (Supplementary Fig. 1)^5^,^17^,^18^,^42^. Spectroscopic analysis was halted at n = 118, once the spot test met both criteria a) and b) producing at least 3–5 spectra per criterion for validation. FT-IR spectroscopy (FT-IR spectrometer Excalibur 3100, VARIAN, Palo Alto, CA, USA) was performed^43^ and the software Resolutions pro (Molecular Spectroscopy Solutions FTIR, Version 4.0, Bio-Rad, Cambridge, MA, USA) was used. The IR spectra were recorded with a resolution of 4 cm^−1^, with 32 scans, with a gain range radius of 40, and a sensitivity of 1, and with a MCT detector. The spectrometer was equipped with a Golden Gate single reflection attenuated total reflection (ATR) unit (Specac Ltd, Orpington, UK) with a diamond crystal. The experimental set-up was at room temperature, and the blow-dried sample was prepared on the crystal. KnowItAll Informatics Systems 7.5 software (Bio-Rad, Cambridge MA, USA) was used to best match spectra of the unknown debris by comparing to the database containing over 90,000 reference IR-spectrums.

Particles were subsequently photographed with a Zeiss Stereomicroscope SV8 with an Axio Cam MRC (Carl Zeiss Microscopy GmbH, Germany) and processed with the software AxioVision, Version 4.5 (Carl Zeiss Imaging Solutions GmbH, Germany).

To prevent samples from being contaminated by airborne particles such as textile fibres, the following measures were taken:

For all procedures, glassware was used, as far as possible. If plasticware had to be utilised, it was rinsed thoroughly with ethanol before first use.

All containers were sealed with Schott lids, glass lids or Parafilm. Labcoats (100% cotton) were worn at all times during transfer procedures. Lab gloves were worn for sorting and counting when hands came in close contact with microscopic samples.

Blanks were run for processes involving plastic utensils at the laboratory (i.e. funnels or pipettes). For all other processes in the laboratory blanks were not run but precautionary measures avoiding contamination in the laboratory were taken as described.

## Additional Information

**How to cite this article**: Mani, T. *et al.* Microplastics Profile along the Rhine River. *Sci. Rep.* **5**, 17988; doi: 10.1038/srep17988 (2015).

## Supplementary Material

Supplementary Information
