# Supplementary material for: Microplastics profile along the Rhine River
Source: Sci Rep. 2015 Dec 8;5:17988. doi: 10.1038/srep17988 (PMC4672315; doi:10.1038/srep17988)
Supplement: Supplementary Information [file srep17988-s1.pdf]

Microplastics profile along the Rhine River

Thomas Mani[1], Armin Hauk[2], Ulrich Walter[2] & Patricia Burkhardt-Holm[1,3]

[1]Man-Society-Environment (Programme MGU), Department of Environmental Sciences, University of Basel, Vesalgasse 1, 4051 Basel, Switzerland, [2]Intertek Switzerland, 4002 Basel,[3] Department of Biological Sciences, University of Alberta, Edmonton, Canada; Corresponding Author: Patricia.Holm@unibas.ch

Additional Information accompanies this paper at http://www.nature.com/srep

Competing financial interests: The authors declare no competing financial interests.

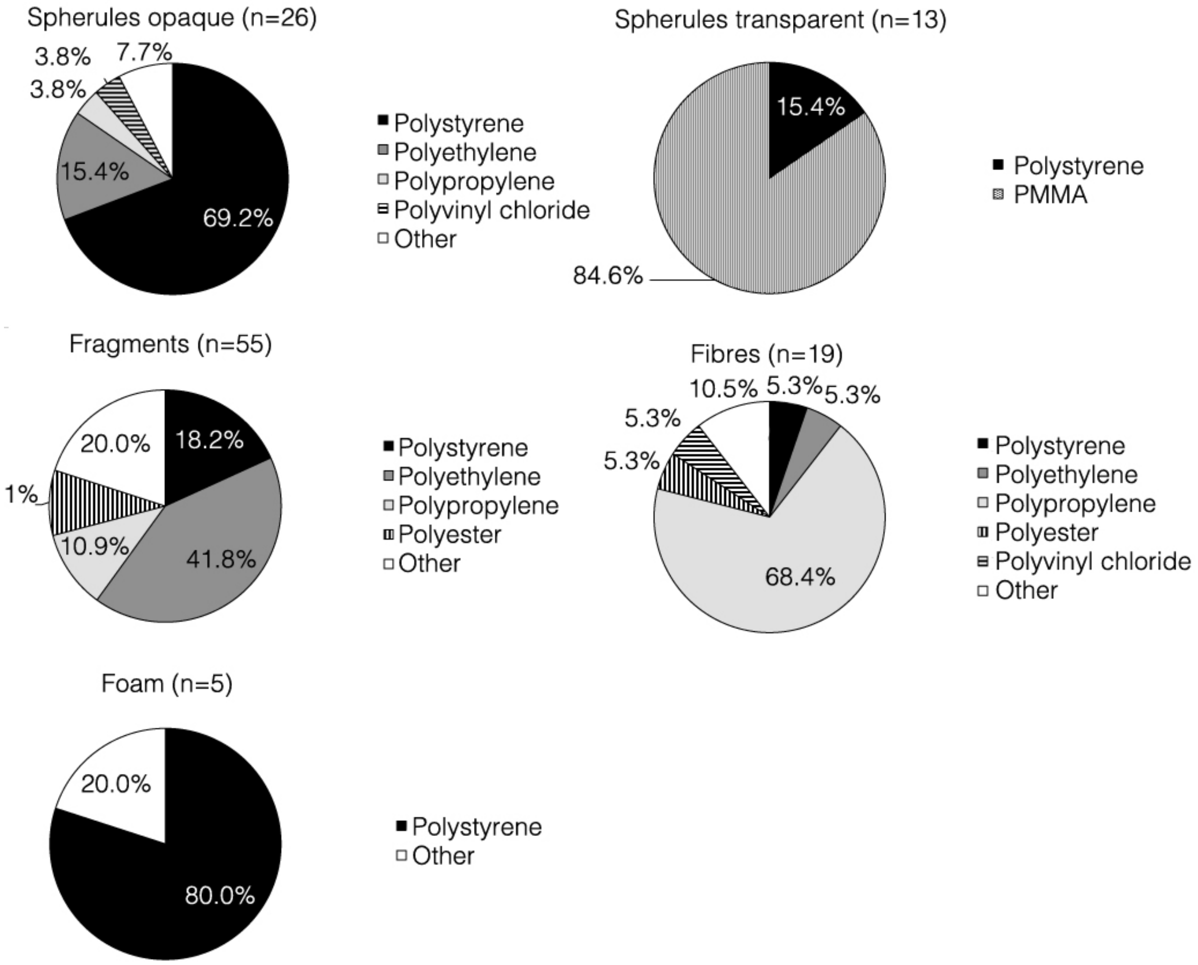

Supplementary Figure 1 | Polymer types of different microplastic categories. Identification of polymer types by Fourier-Transform Infrared Micro-spectroscopy (FT-IR)) (n = 118).

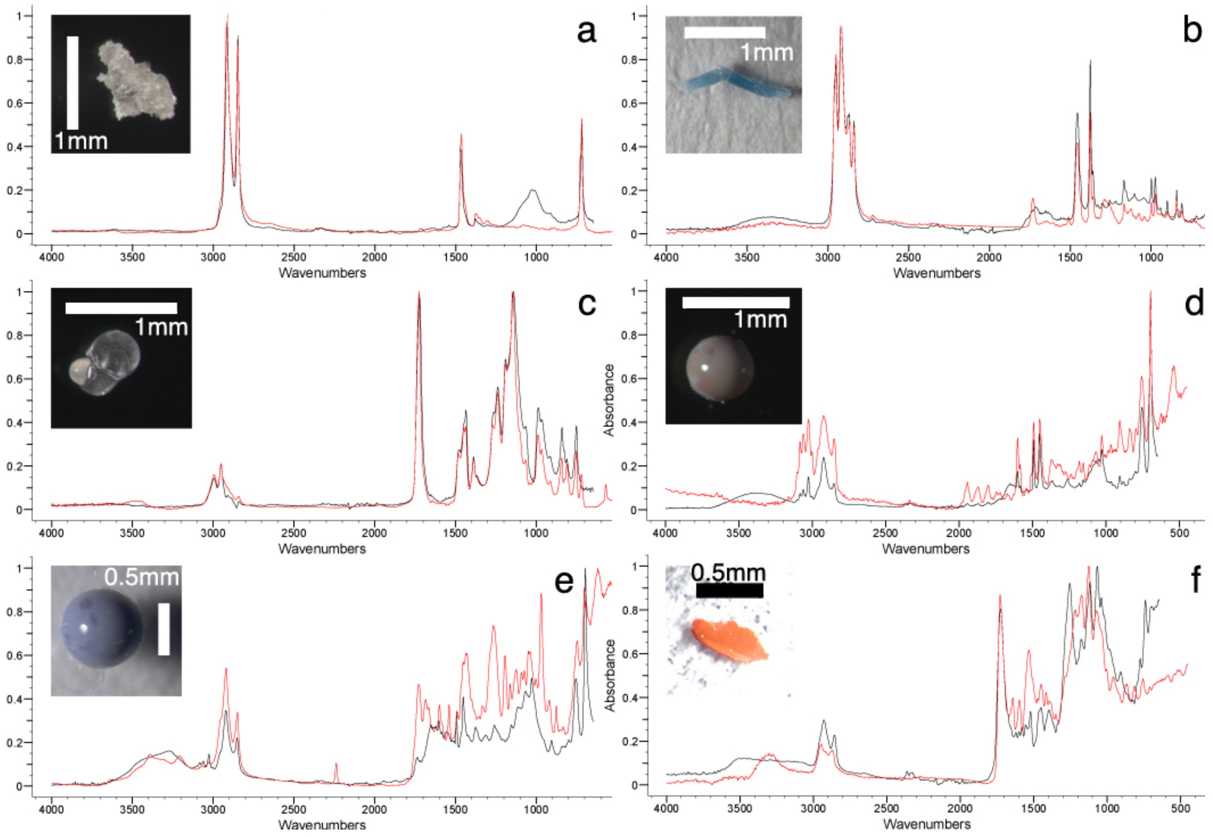

Supplementary Figure 2 | Fourier-Transform Infrared Micro-spectra of selected microplastic particles.

The black curve indicates the recorded spectrum, the red line the reference spectrum from the database (Methods). (a) White fragment, Mainz; polyethylene. (b) Blue fibre, Rees; polypropylene. (c) Clear, colorless spherules clotted (note gas bubble inside), Bad Honnef; polymethyl methacrylate. (d) Opaque, purple spherule, Duisburg; polystyrene (e) Blue spherule, Rees; cross-linked polymer, modified polyvinyl chloride. (f) Red fragment, Cologne-Porz; polyurethane.

Supplementary Table 1 | Characterisation of sampling sites on the Rhine.

Site	River Width (m)	Distance to left shore (m)	Rhine Km	Surface speed (m/s)	Discharge (m^3/s)	Discharge Annual Ø 2014 (m^3/s)	Difference to Annual Ø (%)	WWTP[1] (10 km)	WWTP closest[2] (km)	Inflow[3] (10 km)	Closest Inflow[4] (km)
Basel 1	187	97	165.7	1.73	1,039	1,018	+0.0	2	3	1	1.20
Basel 2	187	97	165.7	1.48	813	1,018	-21.5	2	3	1	1.20
Basel 3	195	135	170.4	0.95	895	1,018	-12.5	3	0.2	3	0.26
Strassbourg/Kehl1 1	244	196	294.2	0.58	1,240	1,170	+5.9	-	-	-	-
Strassbourg/Kehl 2	244	124	294.2	1.18	1,260	1,170	+7.5	-	-	-	-
Strassbourg/Kehl 3	244	50	294.2	0.81	1,250	1,170	+6.8	1	12.2	-	16
Seltz/Rastatt 1	243	77	340	0.85	1,450	1,200	+20.8	2	†0.73	-	25
Seltz/Rastatt 2	243	77	340	0.91	1,460	1,200	+21.7	2	†0.73	-	25
Seltz/Rastatt 3	243	77	340	0.85	1,490	1,200	+24.1	2	†0.73	-	25
Mainz 1	454	82	504	1.01	1,450	◆2,040	-29.0	-	20		-
Mainz 2	454	224	504	0.89	1,460	◆2,040	-28.5	-	-		-
Mainz 3	454	394	504	0.90	1,460	◆2,040	-28.5	-	-	2	1.53
Bad Honnef 1	321	65	640	1.69	2,680	•1,940	+38.1	1	10	1	10
Bad Honnef 2	321	247	640	1.73	2,675	•1,940	+37.8	1	2.23	-	30
Bad Honnef 3	321	164	640	1.88	2,675	•1,940	+37.8	-	-	-	-
Köln-Porz 1	422	315	677	1.27	2,585	■1,965	+31.5	1	9	-	17
Köln-Porz 3	422	87	677	1.94	2,590	■1’965	+31.8	2	8	-	-
Leverkusen 1	368	298	698	0.99	2,550	❖1,995	+27.8	1	4	-	-
Leverkusen 2	368	198	698	2.33	2,560	❖1,995	+28.3	-	-	-	-
Leverkusen 3	368	100	698	1.73	2,560	❖1,995	+28.3	-	17.5	-	-
Duisburg 1	320	240	779.3	1.71	2,710	2,070	+30.9	2	5	-	-
Duisburg 2	320	170	779.3	1.96	2,710	2,070	+30.9	-	-	-	-
Duisburg 3	320	100	779.3	2.38	2,710	2,070	+30.9	1	2	-	-
Rees 1	419	280	837	1.57	2,730	2,090	+30.6	-	13	-	21
Rees 2	419	177	837	1.85	2,740	2,090	+31.1	-	-	-	-
Rees 3	419	81	837	1.75	2,730	2,090	+30.6	1	5	-	-
Zuilichem 1	359	239	944	1.18	1,842	▽1,700	+8.3	-	-		
Zuilichem 3	359	118	944	1.25	1,842	▽1,700	+8.3	-	>50	-	-
Rotterdam 1	385	195	1,000	T1.69	n.a.	n.a.	n.a.	-	-	-	-
Rotterdam 2	385	195	1,000	T2.57	n.a.	n.a.	n.a.	-	-	-	-
Rotterdam 3	385	195	1,000	T2.33	n.a.	n.a.	n.a.	-	-	-	-

T=Transect (surface speed refers to velocity difference between surface and vessel); †=Questionable if WWTP discharges into Rhine before sampling site; w=2013; □=mean measuring station Andernach/Bonn; n=mean measuring station Cologne/Bonn; v=mean measuring station

---

[1] Number of WWTP within 10 km upstream the respective river side.
[2] Distance to closest WWTP upstream on the respective river side.
[3] Number of tributary inflows of higher stream order number within 10 km upstream on the respective river side.
[4] Distance to closest tributary inflow upstream on the respective river side.

Cologne/Düsseldorf; ∇=Average Discharge Waal[1]; n.a. – not available. Discharge data Kehl–Rees: Data source: German Federal Waterways and Shipping Administration (WSV), communicated by the German Federal Institute of Hydrology (BfG), 2015. In Köln-Porz, Leverkusen and Duisburg there are 7, 17 and 5 plastic manufacturers on the Rhine upstream within 10 km, respectively. The distance to the closest plant varies between 0.5, 0.2 and 1.5 km, respectively. For Rees, the closest plastic manufacturer with respect to the sampling site lies 20 km upstream.

Supplementary Table 2 I Microplastic particles km^{-2} in categories and total (300 µm–5 mm) from the Rhine and lakes.

300µm-5mm	n	Fragments	Fibres	Spherules opaque	Spherules trans-parent	Other (foam, foil, pellets)	Total	Average particles km^{-2}	Median	Standard deviation
Basel–Rotterdam	***31***							***892,777***	***359,026***	***1,063,042***
Basel–Mainz	***12***							***202,900***	***145,679***	***175,502***
Basel 1	1	10,655	2,131	4,262	n.a.	55,406	**72,455**			
Basel 2	1	41,143	3,740	2,494	2,494	2,494	**52,364**			
Basel 3	1	572,438	13,583	3,881	1,940	n.a.	**591,842**	238,887	72,455	305,833
Strsb./Kehl 1	1	428,725	35,727	n.a.	23,818	41,682	**529,951**			
Strsb./Kehl. 2	1	112,740	36,014	n.a.	n.a.	n.a.	**148,754**			
Strsb./Kehl 3	1	130,554	6,026	6,026	n.a.	n.a.	**142,605**	273,770	148,754	221,881
Seltz 1	1	82,290	5,741	n.a.	n.a.	3,827	**91,859**			
Seltz 2	1	107,579	n.a.	4,060	n.a.	n.a.	**111,639**			
Seltz 3	1	221,079	7,623	1,906	n.a.	1,906	**232,514**	145,337	111,639	76,142
Mainz 1	1	142,540	10,559	3,520	3,520	1,760	**161,898**			
Mainz 2	1	69,904	11,038	9,198	3,679	3,679	**97,498**			
Mainz 3	1	186,080	5,755	7,673	n.a.	1,918	**201,427**	153,608	161,898	52,458
Bad Honnef–Leverkusen	***8***							***714,053***	***610,608***	***366,105***
Bad Honnef 1	1	403,482	31,746	14,337	87,046	25,602	**562,212**			
Bad Honnef 2	1	417,117	43,012	57,016	306,086	22,006	**845,237**			
Bad Honnef 3	1	49,182	4,918	5,902	291,155	7,869	**359,026**	588,825	562,212	244,195
Cologne-Porz 1	1	734,033	25,154	8,003	294,985	13,720	**1,075,896**			
Cologne-Porz 3	1	116,987	5,358	16,075	141,099	n.a.	**279,519**	677,708	677,708	563,123
Leverkusen 1	1	1,123,986	40,018	13,919	165,292	26,099	**1,369,315**			
Leverkusen 2	1	440,578	12,678	11,886	164,028	7,924	**637,094**			
Leverkusen 3	1	367,741	6,395	36,241	168,415	5,330	**584,122**	863,510	637,094	438,840
Duisburg–Zuilichem	***8***							***2,333,665***	***2,640,910***	***1,130,270***
Duisburg 1	1	394,210	53,854	857,352	120,632	n.a.	**1,426,048**			
Duisburg 2	1	660,556	n.a.	2,014,341	349,706	50,336	**3,074,940**			
Duisburg 3	1	724,638	n.a.	1,905,960	283,486	3,107	**2,917,191**	2,472,726	2,917,191	909,875
Rees 1	1	422,910	17,670	3,199,510	272,123	18,848	**3,931,062**			
Rees 2	1	1,112,712	38,024	1,454,931	n.a.	5,003	**2,610,671**			
Rees 3	1	922,454	117,043	1,312,266	309,469	9,919	**2,671,150**	3,070,961	2,671,150	745,483
Zuilichem 1	1	123,980	51,789	70,621	54,928	n.a.	**301,318**			
Zuilichem 3	1	927,652	136,115	418,701	247,078	7,398	**1,736,943**	1,019,131	1,019,131	1,015,140
Rotterdam	***3***							***286,517***	***272,599***	***41,787***
Rotterdam 1	1	120,274	38,269	76,538	91,845	6,560	**333,486**			
Rotterdam 2	1	112,924	6,473	82,715	56,102	14,385	**272,599**			
Rotterdam 3	1	107,265	26,220	44,495	74,689	795	**253,464**	286,517	272,599	41,787
Lake Geneva[A]	4	n.a.	n.a.	n.a.	n.a.	n.a.	n.a.	220,000	220,000	160,000
Lake Geneva[B]	4	n.a.	n.a.	n.a.	n.a.	n.a.	n.a.	33,000	14,000	46,000
Lake Constance	3	n.a.	n.a.	n.a.	n.a.	n.a.	n.a.	61,000	63,000	12,000
Lake Neuchâtel	3	n.a.	n.a.	n.a.	n.a.	n.a.	n.a.	61,000	62,000	24,000
Lake Maggiore	4	n.a.	n.a.	n.a.	n.a.	n.a.	n.a.	220,000	220,000	150,000
Lake Zurich	5	n.a.	n.a.	n.a.	n.a.	n.a.	n.a.	11,000	9,800	2,600
Lake Brienz	4	n.a.	n.a.	n.a.	n.a.	n.a.	n.a.	36,000	30,000	23,000
Lake Erie (b) (USA)	8	n.a.	n.a.	n.a.	n.a.	n.a.	n.a.	105,503	n.a.	n.a.
Lake Superior (USA)	5	n.a.	n.a.	n.a.	n.a.	n.a.	n.a.	5,390	n.a.	n.a.
Lake Huron (USA)	8	n.a.	n.a.	n.a.	n.a.	n.a.	n.a.	2,779	n.a.	n.a.
Lake Khövsgöl (Mongolia)	8	n.a.	n.a.	n.a.	n.a.	n.a.	n.a.	20,000	n.a.	n.a.

Bold, italicised rows summarize the subsequent rows. The table is complemented with data from Swiss lakes (CH;[2]), Lakes Erie (b), Superior and Huron[3] and Lake Khövsgöl in Mongolia[4]. Lake Geneva[A,B] refers to Grand Lac part, and Petite Lac part of this lake, respectively. The weighted average microplastic concentration in the Rhine (892,777 particles km^{-2}) is calculated by taking the mean concentration of all sampling sites between Basel and Rotterdam as opposed to dividing the total particles by the total sampled area. n.a. = not available.

Supplementary Table 3 | Microplastic particles 1,000m^{-3} from the Rhine and other rivers.

**Sampling Site**	**n**	**Microplastics** average	**1,000m^{-3}** median	std. dev.	max.
Rhine (Basel–Rotterdam)	*31*	*4,960*	*1,995*	*5,906*	*21,839.0*
Basel-Mainz	*12*	*1,127*	*809*	*975*	
Bad Honnef–Leverkusen	*8*	*3,967*	*3,392*	*2,034*	
Duisburg–Zuilichem	*8*	*11,885*	*14,672*	*6,279*	
Rotterdam	*3*	*1,592*	*1,514*	*232*	
Basel*	3	1,327	403	1,699	
Strassbourg/Kehl*	3	1,521	826	1,233	
Seltz°	3	807	620	423	
Mainz°	3	853	899	291	
Bad Honnef*	3	3,271	3,123	1,357	
Cologne Porz°	2	3,765	3,765	3,128	
Leverkusen*	3	4,797	3,539	2,438	
Duisburg*	3	13,737	16,207	5,055	
Rees*	3	17,061	14,840	4,142	
Zuilichem*	2	5,662	5,662	5,640	
Rotterdam*	3	1,592	1,514	232	
Rhône♮ (CH)	4	2,300	2,400	530	
Aubonne (CH)	4	100	100	42	
Venoge* (CH)	3	6,500	4,000	5,300	
Venoge° (CH)	2	64,000	-	35,000	
Vuachère (CH)	2	4,400	-	1,300	
Rhône, Geneva ♭ (CH)	4	130	120	76	
Rhône, Chancy ♭ (CH)	5	290	250	80	
Danube 2010 (AT)	951	*937.6*	-	*8,544*	*141,648*
Danube 2012 (AT)		*55.1*	-	*75*	*745*
Coyote Creek° (USA)	1	<2,000	-	-	-
Coyote Creek* (USA)	1	<6,000	-	-	-
San Gabriel River° (USA)	1	171,000	-	-	-
San Gabriel River* (USA)	1	<1,000	-	-	-
Los Angeles River° (USA)	1	<10,000	-	-	-
Los Angeles River* (USA)	1	<1,000	-	-	-
North Shore Channel○ (USA)	4	1,940	-	810	-
North Shore Channel⊙ (USA)	4	17,930	-	11,050	-

Data from the Rhine (average/sample location and in groups) compared with data from the Danube (500 µm mesh)[5], Coyote Creek, San Gabriel River and Los Angeles River (333 µm mesh)[6], Swiss rivers (300 µm)[2] and the North Shore Channel, Chicago (333 µm)[7].

*=dry, °=wet/rainy, ♮=upstream Lake Geneva, ♭=downstream Lake Geneva, ○=upstream Waste Water Treatment Plant (WWTP), ⊙=downstream WWTP (definition of specifications according to respective papers)

Supplementary References

1. Schielen, R. M. J., Jesse P. & Bolwidt, L. J. On the use of flexible spillways to control the discharge ratio of the Rhine in the Netherlands: hydraulic and morphological observations. *Neth. Journ. Geosc.* **86**, 77–88 (2007).
2. Faure, F., Demars, C., Wieser, O., Kunz, M. & Alencastro, L. Plastic pollution in Swiss surface waters: nature and concentrations, interaction with pollutants. *Environ. Chem.* [in press].
3. Eriksen, M., *et al.*, Microplastic pollution in the surface waters of the Laurentian Great Lakes. *Mar. Pollut. Bull.* **77**, 177–182 (2013).
4. Free, C. M., Jensen, O.P., Mason, S. A., Eriksen, M., Williamson, N. J., Boldgiv, B., High-levels of microplastic pollution in a large, remote, mountain lake. *Mar. Pollut. Bull.* **85**, 156–163 (2014).
5. Lechner, A., *et al.*, The Danube so colourful: A potpourri of plastic litter outnumbers fish larvae in Europe's second largest river. *Environ. Pollut.* **188**, 177–81 (2014).
6. Moore, C. J., Lattin, G. L. & Zellers, A. F. Quantity and type of plastic debris flowing from two urban rivers to coastal waters and beaches of Southern California. *Rev. Gestão Costeira Integr.* **11**, 65–73 (2011).
7. McCormick, T. J., Hoellein, S. A., Mason, J. & Schluep, Kelly J. J. Microplastic is an abundant and distinct microbial habitat in an urban river. *Environ. Sci. Technol.* **48**, 11863–11871 (2014).
